# The Common Task

**Published:** 1907-04-06

**Authors:** 


### THE COMMON TASK.

### Contracts for Meat.

Two systems are in vogue with regard to contracts for meat in institutions. The first is known as the all-round system, and under this one price is agreed upon, for which every joint is to be supplied. Under the second system every joint is contracted for under its own price, and, where matters are very carefully regulated, at different prices according to the season of the year. It is a moot point which system yields the best results, and this can really only be settled by trying both ways, and watching the bills. A good deal of light has been thrown on the matter by an experienced steward who has lately advised his committee to return to the "all-round" system. He discovered that the tendency in catering for a large body of officials was always towards the more expensive joints, and that in the absence of any efficient check the adoption of the all-round price made a difference of something like a fifth of the cost. Where the reins are in the hands of an experienced housekeeper or steward armed with full responsibility, better results should be obtainable by clearly defining what is obtained for the money, because it is possible to steer a course by market prices, and give the household the value of a cheap season for certain delicacies. But when there is no one of sufficient authority to resist the steady pressure in favour of extravagance, which must always be looked for on the part of those who are catered for officially, it will no doubt often remedy matters to leave the choice in the hands of the contractor.

### Convalescents.

The little valedictory service held every fortnight in the chapel at the Middlesex Hospital for patients going down to the Clacton Home the following morning is a very interesting feature of hospital life. Entirely simple and devotional in character, it forms an object-lesson in the benefits conferred in the modern hospital outside the gift of healing by the introduction to higher ideals of life and conduct. There can be no two opinions about the value of the convalescent period in building up moral as well as physical strength, and though the bracing and civilising effects of hospital sojourn are mainly hidden from view, all who know the lives of the poor are aware that they are far-reaching. It is well that periodically there should be a reminder that what constitutes a monotonous routine for the staff is in the nature of a great crisis to nine out of ten patients, the most striking event probably which has marked the dead level of their lives.

### The Average Cost of Nursing Salaries per Bed.

The figures available this year in "Burdett's Annual" as to the cost of the nursing from the point of view of salaries are very interesting. The upward tendency is still observable, and is perhaps likely to continue, the maximum among general hospitals with medical schools being touched by the London Hospital with an average rate of £12 7s. per bed. The minimum is £4 1s. at the Radcliffe Infirmary, and the difference in rate between the London and the provincial hospitals points to a wide difference in general organisation. Out of a staff of 58 the Meath General Hospital shows a rate of £1 7s. per bed with 38 first year probationers (25 of whom are paying pupils). Of the general hospitals without medical schools the highest rate is at the Poplar Hospital, with a cost of £10 4s. per bed, and the lowest at the Essex and Colchester Infirmary, which works out at £2 4s. The inference which may be drawn from these widely varying figures is that hospital managers have not yet come to general agreement as to the number of nurses required to ensure that the nursing is done under the best conditions. So far as we have been able to ascertain the salaries tend to be much the same in one hospital as in another; the difference in cost appears to lie in the numbers.

### Beef-tea Extracts.

"Lady Guardian" asks what form of concentrated beef-tea is the most economical to use in large quantities. We are disposed to think that no prepared extract is really economical unless it is a free gift, but as its use may be a matter of great convenience occasionally, it would be a useful experiment to work out the comparative result obtained from 4 oz. of several advertised varieties, comparing the cost on the basis of the price in bulk. The price of freshly made beef-tea should not be more than 2d. a pint at the outside. We should be glad to have some details of practical experience on this subject.

Correspondence and queries for this section to be sent to the office of The Hospital, 28 Southampton Street, Strand, and marked *Nursing Administration*.

